# EXPLORING THE UTILIZATION OF MERIT-BASED INCENTIVE PAYMENT SYSTEM (MIPS) PROGRAMS IN WORKFORCE DEVELOPMENT

**DOI:** 10.1093/geroni/igad104.1702

**Published:** 2023-12-21

**Authors:** Sam Cotton, Anna Faul

## Abstract

Measuring patient outcomes is a critical component of the HRSA funded Geriatric Workforce Enhancement Programs (GWEPs), which aim to improve the quality of care for older adult patients. In 2019, GWEPs were tasked with aligning patient outcomes with the CMS Merit-based Incentive Payment System (MIPS) program, which incentivizes quality care and patient outcomes. Collecting and using MIPS data effectively can be a challenging task, particularly when done across multiple levels of care. To address these challenges, the National Association of Geriatric Education Centers (NAGEC) created a workgroup to develop advocacy and solutions for patient outcomes. This symposium will discuss the utilization of MIPS as performance metrics across three GWEPs, the challenges in reporting these outcomes, and implications for future policy changes. This symposium’s primary objective is to explore how GWEPs can effectively measure patient outcomes, despite the challenges involved in collecting and utilizing MIPS data. By discussing these challenges and proposing potential solutions, this symposium aims to improve the effectiveness and sustainability of GWEPs, ultimately leading to better outcomes for older adult patients. Overall, measuring patient outcomes is critical for ensuring that older adult patients receive high-quality care that is tailored to their individual needs and preferences. Despite the challenges involved in collecting and utilizing MIPS data, our symposium provides an opportunity to explore potential solutions and advocate for policies that support improved patient outcomes in geriatric care.

